# Sharing EHR data of patients with rare diseases for research - The role of quality assessments in a national federated research data infrastructure

**DOI:** 10.1186/s12911-025-03153-x

**Published:** 2026-03-02

**Authors:** Kais Tahar, Raphael Verbuecheln, Tamara Martin, Holm Graessner, Stephanie Biergans, Dagmar Krefting

**Affiliations:** 1 Department of Medical Informatics, University Medical Center Göttingen, Georg-August-University, 37075 Göttingen, Germany; 2 Institute for Public Health in Acute Medicine, University Medicine Magdeburg, Magdeburg, Germany; 3 Medical Data Integration Center, University Hospital Tübingen, Tübingen, Germany; 4 Centre for Rare Diseases, University Hospital Tübingen, Tübingen, Germany

**Keywords:** Data quality, Rare Diseases, FAIR guiding principles, EHR data, Healthcare standards, Semantic annotation, Interoperability, Medical data integration, Hospital information system, ETL, HL7 FHIR

## Abstract

**Background:**

To obtain sufficient data for clinical research on Rare Diseases (RDs), the project “Collaboration on Rare Diseases” (CORD-MI) has connected 24 University Medical Centers (UMCs) in Germany. As part of the national Medical Informatics Initiative (MII), which aims at making the EHR data of all German UMCs FAIR and jointly usable for research, CORD-MI extends the methods and tools along the special requirements of RD data. Major challenges are insufficient coding of RDs and the high influence of individual data records on the results due to low cohort sizes. Hence, high data quality (DQ) of each data record is vital and exclusion due to quality issues may lead to insufficient sample sizes. DQ deficiencies induced by a FAIRification process may be corrected retrospectively; therefore their identification is of high relevance for RD research.

**Methods:**

We used a self-developed DQ assessment tool to investigate the impact of the implemented FAIRification processes. Seven normalized DQ indicators – encompassing completeness, uniqueness, and plausibility – were assessed automatically across two datasets created from the same patient information system (PAS) but processed differently: claims data available in a FAIR format by MII standards (FHIR-DS), and a direct export (EXP-DS). The results were independently validated by domain experts, and measures to resolve the DQ issues were investigated. Furthermore, the tool’s FAIRness and computational performance were evaluated.

**Results:**

We found a higher number of DQ issues in FHIR-DS than in EXP-DS. Specifically, FHIR-DS contained 1988 distinct DQ issues: nine ambiguous RD cases, two missing data items, and 1977 completeness issues induced by the FAIRification processes. We could successfully resolve the majority (68%) of all identified DQ issues in FHIR-DS. The runtime performance could be reduced from 12.52 min to 0.12 min for FHIR-DS by multi-core parallelization. A comparison with current FAIR guidelines of the software showed compliance with 16 of 17 principles.

**Conclusion:**

Completeness indicators are crucial for evaluating the quality of RD data, as most identified issues are related to the completeness dimension and largely stem from FAIRification processes. Our findings have shown the efficiency and effectiveness of our methodology in improving the quality and usefulness of FAIR data both retrospectively and prospectively.

**Clinical trial number:**

Not applicable.

**Supplementary Information:**

The online version contains supplementary material available at 10.1186/s12911-025-03153-x.

## Introduction

With the increasing digitization of healthcare and hospital services, there is also an increasing focus on the contents of the electronic health record (EHR). This growing repository of data collected in the hospital information system (HIS) represents a valuable source of information for clinical researchers [1]. The secondary use of EHR data is considered to improve the quality of healthcare and clinical research [2]. However, as EHR data is mainly collected for clinical care and billing purposes [1, 3], it shows completely different characteristics than data captured for research – for example, medical registries and clinical trials. They typically have a high degree of structured data and quality control is a main effort in such research data collections [4]. Previous studies have indicated that the quality of EHR data may not be sufficient for clinical research [1, 5]. Therefore, there is a need to develop useful methods to assess and improve the data quality (DQ) of EHRs. According to the widely used definition of DQ as “fitness for use” [6], useful DQ assessments mainly depend on the domain-specific context - especially in the field of Rare Diseases (RDs).

In Europe, RDs are defined as diseases that affect less than 5 in 10,000 people [7]. To improve the visibility of RDs in EHR data and support clinical research, the research project “Collaboration on Rare Diseases” (CORD-MI) [8] of the Medical Informatics Initiative (MII) [2] has connected 24 University Medical Centers (UMCs) in Germany. Each participating hospital of the MII has committed to making EHR data of their inpatient cases available and reusable for clinical research. The MII has taken several measures to make this data FAIR, i.e. findable, accessible, interoperable, and reusable according to the respective guiding principles, first introduced by Wilkinson et al. [9]. The CORD-MI consortium as part of the MII builds upon the generic FAIRification measures and adopts, improves and extends them for the specific needs of RD data sharing.

A major pillar of the MII FAIRification efforts is the MII Core Data Set (MII-CDS). Defined and implemented as Fast Healthcare Interoperability Resources (FHIR) [10], UMCs are required to provide part of their EHR data in alignment with the FHIR standard specification of the MII-CDS (for details, see the “Methods” section). We would like to underscore that the FAIRification processes mentioned above go beyond simply converting data into the FHIR format. They also enhance accessibility, improve semantic interoperability, and incorporate metadata [11]. The findability is, for instance, ensured by the German Portal for Medical Research Data (FDPG), as reported by a FAIRness assessment including the MII infrastructure [11]. The connected infrastructure of the MII allows now to find and apply for the clinical routine data from all 41 participating hospitals using the FDPG. Currently, this central portal contains clinical data from more than 21 M persons, encompassing more than 250 M diagnoses, 100 M procedures, and 2 Bn lab values [12]. More than 100 data requests have been submitted and documented in the national project registry of the FDPG.

The transformation of EHR data stored in the different subsystems of the HIS to MII-CDS implies the application of extract, transform, and load (ETL) processes. Due to the heterogeneity of the underlying information systems, documentation policies, and relevance for the primary usage context, ETL pipelines are highly heterogeneous across UMCs and are typically individual software solutions of the local medical data integration centers (meDICs) that are responsible for this task at each participating UMCs. A pragmatic solution has been to use claims data as input for ETL processes as it is already highly structured and standardized. This approach was for example successful in monitoring hospital admission rates during the COVID−19 pandemic [13]. The claims data is provided by the patient administration system (PAS), a subsystem of the HIS that is responsible for patient admission, patient discharge, and medical billing. However, as claims data is compiled for a specific primary goal, its usage as a data source and the following ETL processes raise concerns about the influence on the quality of transformed data, such as completeness, plausibility, and semantic integrity [1, 14].

A high DQ is required to support the proper reuse of EHR data for clinical research on RDs and ensure a high evidence level of scientific outcomes derived from this data [15]. Due to the overall low number of datasets in RDs, DQ issues may have a higher impact than in common diseases, where sufficient case numbers are typically still reached after the removal of low-quality data. Furthermore, RD documentation shows some special characteristics. Most RDs are not represented in the coding scheme for claims data, the International Classification of Diseases and Related Health Problems, 10th revision, German Modification (ICD-10-GM) [16]. RDs are often subsumed in unspecified ICD-10-GM codes, which make the majority of RDs invisible or indistinguishable in HISs [17]. To unambiguously identify RDs, Orphacodes are used. Orphacodes are specific identification numbers assigned to each RD entity in the Orphanet Classification [18]. In Germany, specific documentation standards for RDs have been recently enforced. Since 2023 it has been mandatory in Germany to document certain RDs with a special code in claims data, which we will describe in more detail in the “Methods” section.

To summarize, RD data reuse strongly relies on high-quality data, and therefore specific attention is required to avoid introducing any DQ issues by the FAIRification processes. Thus specific DQ assessments to detect and mitigate potential DQ issues are necessary. Various metrics and frameworks for DQ assessments have been proposed in previous works [19–21], that we have adopted and extended for application on RD in previous studies [22, 23]. In this paper, we apply these methods to investigate the influence of the FAIRification processes, in particular the transformation of the clinical data to claims data and further to the MII-CDS on the example of one UMC participating in CORD-MI. Evaluating the impact of FAIRification processes on DQ involves assessing the quality of the transformed dataset. Specifically, this impact is evaluated by a comparative analysis of DQ issues identified in two datasets originating from the same data source but processed differently. Given the high importance of conducting and frequently repeating such DQ assessments at all participating sites, this study also optimizes and evaluates the computational performance and FAIRness of the developed software. The FAIR principles should not be limited to data but should also encompass all digital artifacts, including software tools. In this context, we describe the measures implemented to ensure that our tools complies with the FAIR principles. Finally, we discuss the results and insights gained from this study and conclude with recommendations for ensuring high-quality FAIR data to support clinical research on RDs.

## Methods

In this section, we present the analyzed datasets and give more details about the specific technical and organizational context of the FAIRification processes, the national RD coding guidelines, and the employed methods and tools for DQ assessments. Since the terms used in previous studies on DQ are often ambiguous, we follow in this paper the terminology presented in [23].

### Technical and organizational frame of the medical informatics initiative: components and standards

As described above, the MII is a national research data infrastructure to enable the secondary use of EHR data from all hospitals that sign the legally binding participation contract. The main organizational components are the meDICs that are set up at the sites. They are responsible, among other things, for transforming EHR data into MII-CDS FHIR format (i.e. contributing to data FAIRification), organizing local Use and Access processes, and data provisioning. Available data can be found and applied for on the central Research Health Data Portal [24]. Data sharing processes within the MII rely on the MII-CDS. It has been developed as a modular national information model that defines a FHIR standard to harmonize, exchange, and federate health data for research in Germany [25]. The MII-CDS defines base modules that apply to all analyzed cases and are expected to be relevant for the majority of clinical studies in the CORD-MI research network. These base modules, in turn, contain mandatory data items, that are expected to be available at most meDICs.

### Documentation of RD in Germany

In Germany, the coding system ICD-10-GM is used in claims data to describe diagnoses. Some ICD-10-GM codes exclusively code a single RD, some code only RDs, but more than one, and some may encompass both rare and common diseases. This has led to attempts to define the so-called tracer diagnoses, i.e. ICD-10-GM codes that exclusively code RD cases, regardless of the number of RDs encompassed by the code [26]. Within CORD-MI, a list of 143 tracer diagnoses has been defined as a reference, which we refer to as CORD-MI Tracers [27].

In addition to ICD-10-GM, the Federal Institute of Drugs and Medical Devices (BfArM) provides an alphabetical list of medical and natural language diagnostic terms based on the alphabetical index of ICD-10-GM. This coding framework, known as Alpha-ID, provides unique identifiers (IDs) for more than 89.000 diagnostic terms, enabling differentiated diagnostic coding for diseases listed in the ICD-10-GM [28]. For example, the ICD-GM-10 code Q87.5 represents more than 20 Alpha-IDs, including I128560 and I128737, which are assigned to the diagnostic terms “Grant syndrome” and “Lowry-Wood syndrome” respectively.

The Alpha-ID system is a stable and maintained terminology, even if a diagnostic term is removed from ICD-10-GM. Since 2015, the Alpha-ID system has been extended to incorporate an additional field for Orphacodes [28], bridging the gap between the ICD-10-GM and Orphanet classification systems. As of 2023, it is mandatory to document the Orphacode in claims data for RD patients if the code is included in the Alpha-ID-SE terminology, making it the gold standard of RD coding in Germany for billing purposes. This coding system enables a uniform and standardized approach to document RDs according to both ICD-10-GM and Orphanet systems. It can be used to define an automatically generated tracer list that includes ICD-10-GM codes, where all diagnostic terms associated with this code also have an associated Orphacode. We refer to these ICD-10-GM codes as Alpha-ID-SE Tracers. For instance, the ICD-10-GM code Q87.5 represents an Alpha-ID-SE Tracer, which legally requires the use of Orphacode 2097 for documenting patients with Grant syndrome, and Orphacode 1824 for the documentation of Lowry-Wood syndrome. We would like to emphasize that such Alpha-ID-SE Tracers may differ strongly from the above-mentioned CORD-MI Tracers, which have been generated by clinical experts.

### Data

The study population comprised inpatient cases from 2021, that included Orphacodes or CORD-MI Tracers [27]. The study cohort comprises a total of 1424 cases from 970 different patients that have been seen in the University Hospital Tübingen (UKT) and were documented in the PAS. UKT uses the hospital information system SAP-IS/H [29] as PAS, which was the most common PAS installed in CORD-MI hospitals.

To investigate the impact of FAIRification processes, in particular data transformation, on DQ, two datasets were used in this study:

(1) As part of the MII the meDIC at UKT has developed ETL-pipelines to provide pseudonymised data in a FHIR format that is compliant with the MII-CDS specification. Data for the MII-CDS profiles (Person v1.0.14, Encounter v1.0.0, Condition v1.0.4) was derived from PAS claims data according to the German § 21 Hospital Remuneration Act (KHEntgG) from 2021 [30], and was stored by the meDIC on a FHIR server (IBM). The study population of RD cases was applied for through the data use and access committee at UKT and provided by the meDIC for local analysis. This study investigated 15 mandatory data items specified by the FHIR profiles Patient, Encounter, and Condition, which are defined in the MII-CDS modules Person, Treatment Case, and Diagnosis, respectively. We will refer to this dataset as FHIR-DS from hereafter. It is important to note that in 2021, Orphacodes were not included into the claims data.

(2) Additionally, a second dataset was used, which had been exported directly from the PAS and provided to the meDIC in a pseudonymized version (CSV formatted table). This data was extracted for quality control purposes as part of the CORD-MI project, based on the CORD-MI study protocol, and used accordingly in this study. The data defined in the study protocol was generally aligned with the FHIR-DS but was not identical to it, e.g., both inpatients and outpatients were included. The CORD-MI Tracers were used as a basis for filtering RD diagnoses if no Orphacode was present, therefore this dataset solely contained RD patients diagnosed with Orphacodes or ICD-10-GM codes listed in the CORD-MI Tracers. This dataset was stored and processed by the meDIC on a dedicated project virtual machine. For this study, the dataset was additionally filtered to only include inpatients. We refer to this dataset as EXP-DS.

**Fig. 1:**
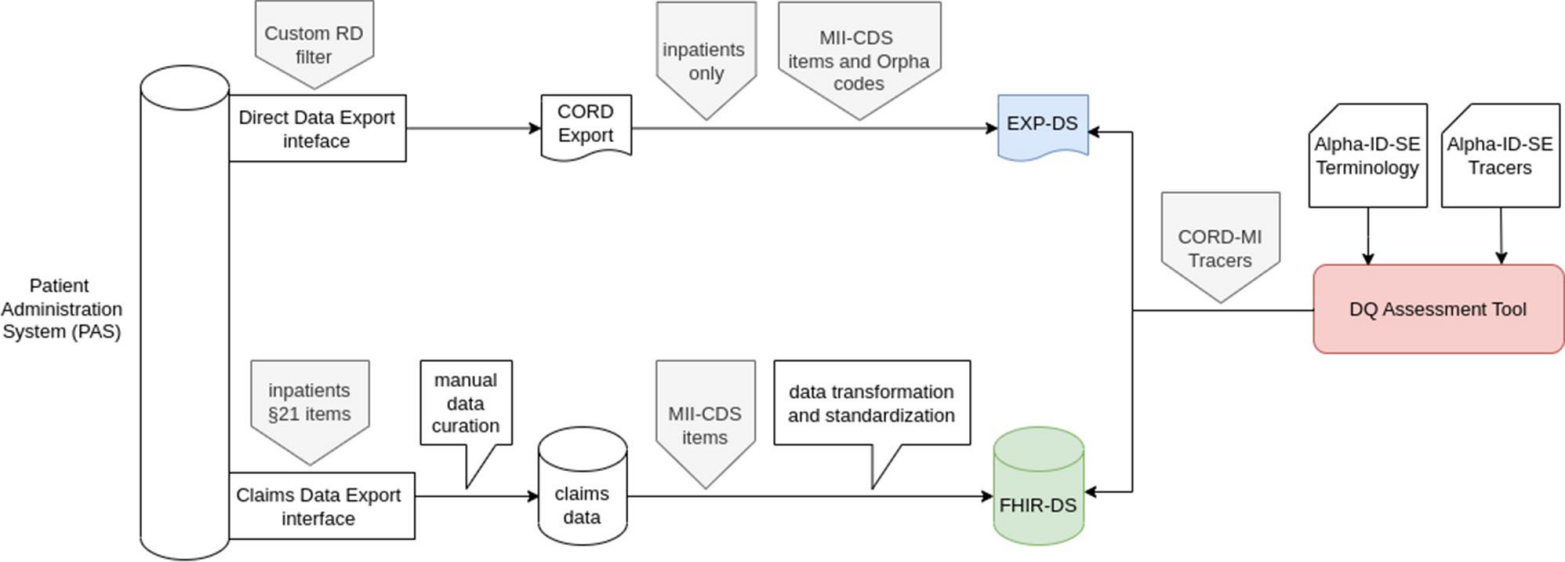
Technical Infrastructure for evaluating the quality of EHR data on RDs

Figure 1 shows the data processing pipelines from the common source database to the respective datasets.

**Fig. 2:**
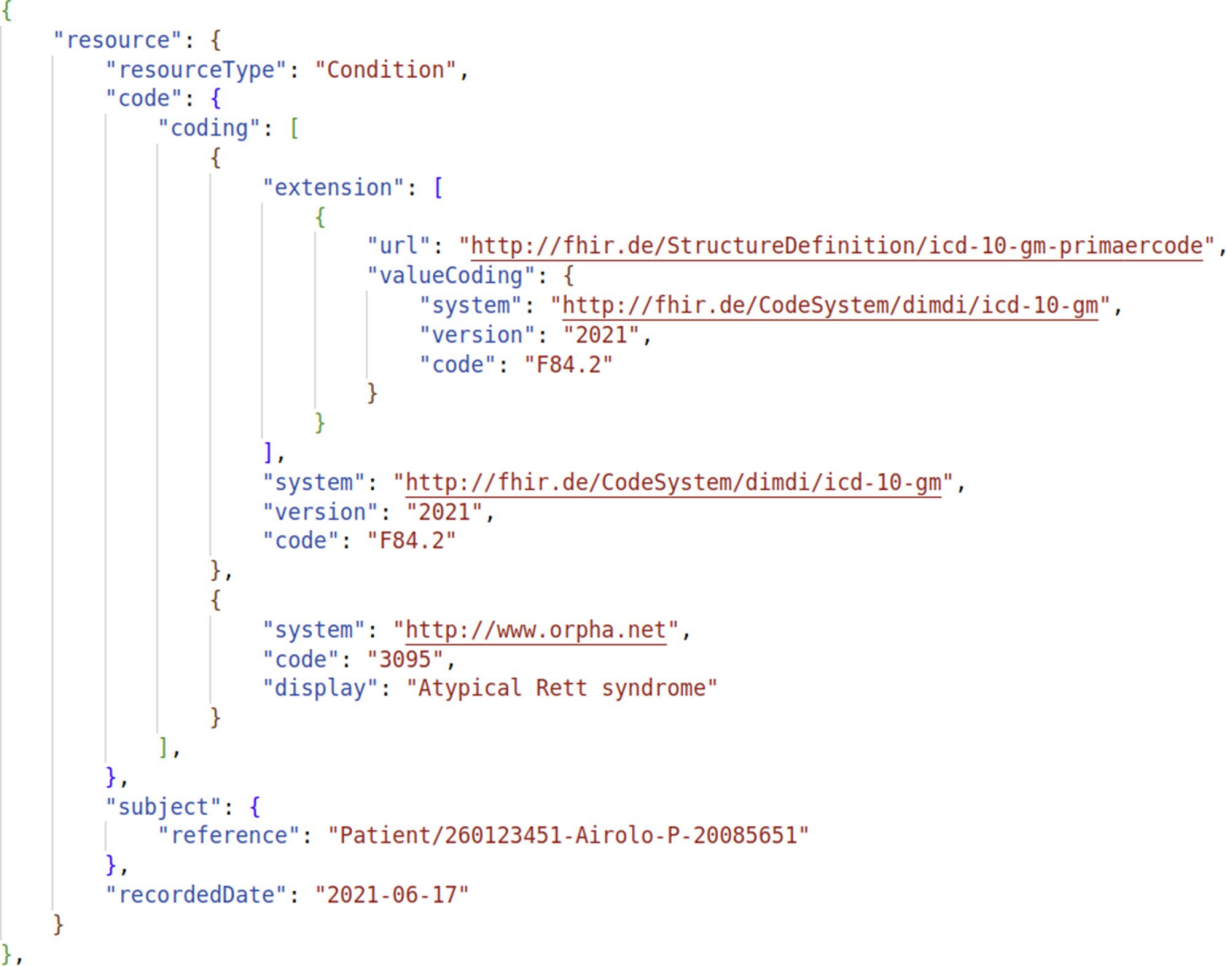
JSON schema representing an exemplary instance of the diagnosis module within the FHIR-DS

As mentioned above, the MII-CDS encompasses multiple data modules that are included in the FHIR-DS. The EXP-DS, however, does not comply with this structure since it is directly derived from the PAS. For instance, Table 1 and Fig. 2 illustrate the divergent structure for diagnostic information across the two datasets. For further details, synthetic datasets are available on GitHub [27], which include examples for both formats.

**Table 1:**
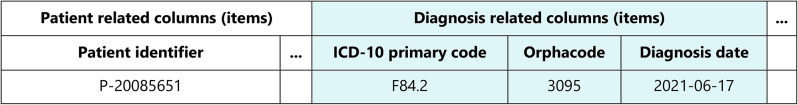
Structure of the CSV table representing the diagnostic information within the EXP-DS (see blue highlighted cells)

### Data quality assessment

The Data Quality Assessment employs the conceptual framework and tools that we have developed within CORD-MI specifically for DQ assessments of RD data [23]. Briefly described, the concept of DQ is often defined according to the ISO 8000−2:2022 standard as the “degree to which a set of inherent characteristics of data fulfills requirements” [31]. Such characteristics are modeled as Data Quality Indicators, where the degree of requirement fulfillment is typically given as a value between 0 as minimum and 100% as optimum. We use different DQ indicators, which we semantically group into the quality dimensions of completeness, uniqueness and plausibility. Table 2 shows the DQ indicators used in our DQ assessments and provides a concrete example for each indicator to illustrate how they can be applied in practice. Two factors were considered for the selection of DQ indicators: (a) the indicators should reflect the use case requirements, which were elicited by a previous study [23], and (b) the indicators should cover different aspects, incorporating both specific and generic characteristics of DQ. Based on these criteria, a total of seven indicators were selected. Please note that four of them are explicitly related to Orphacoding and RD cases.

Furthermore, the DQ indicators were calculated based on absolute quantities of observation units, the so-called DQ parameters listed in Table 3. The first six parameters count observation units like RD cases while the remaining ones count different types of DQ issues, such as missing Orphacodes or missing mandatory data values. It should be noted that in the context of DQ assessment, tracer refers to the Alpha-ID-SE based tracer diagnosis list, which we further employed to distinguish between ambiguous and unambiguous diagnoses.

**Table 2:** DQ dimensions and indicators of dataset assessments used in this study

Dimension	DQ Indicator		
	Name	Abbreviation	‍Definition and examples
Completeness	Orphacoding Completeness Rate	dqi_co_ocr	dqi_co_ocr evaluates whether all tracer diagnoses are coded using Orphacodes. For example, the absence of an Orphacode in a diagnosis coded with the ICD-10-GM code E84.0 will be assessed as a completeness issue
	Item Completeness Rate	dqi_co_icr	‍dqi_co_icr assesses whether all mandatory metadata (e.g. the data item “diagnosis date”) are present in the examined dataset
	Value Completeness Rate	dqi_co_vcr	dqi_co_vcr evaluates the degree to which mandatory data values (e.g. individual birth dates) are complete
Plausibility	Orphacoding Plausibility Rate	dqi_pl_opr	‍dqi_pl_opr assesses the proportion of RD cases where the combination of the ICD-10-GM code and Orphacode is plausible according to Alpha-ID-SE. For example the combination G71.2 and 777 will be assessed as implausible link
	Range Plausibility Rate	dqi_pl_rpr	‍dqi_pl_rpr evaluates the plausibility of data values by identifying outliers, such as age values exceeding 130
Uniqueness	RD Case Unambiguity Rate	dqi_un_cur	‍dqi_un_cur assesses the unambiguity of RD cases by identifying cases with ambiguous RD diagnoses, such as the ICD-10-GM code Q87.5 mentioned above
	RD Case Dissimilarity Rate	dqi_un_cdr	‍dqi_un_cdr assesses the uniqueness of RD cases by identifying duplicates within the analyzed datasets. Duplication are for examples RD cases with identical case ID, patient ID, ICD-10-GM code, and Orphacodes

**Table 3:** Used DQ parameters

No.	Name	Abbreviation	Short description
P1	RD Cases	rdCase	Number of treatment cases documented at least using an Orphacode or an Alpha-ID-SE Tracer
P2	Orpha Cases	orphaCase	Number of treatment cases diagnosed using an Orphacode
P3	Tracer Cases	tracerCase	Number of treatment cases diagnosed using Alpha-ID-SE Tracers
P4	RD Case rel. Frequency	rdCase_rel	Relative frequency of RD cases normalized to 100,000 inpatient cases
P5	Orpha Case rel. Frequency	orphaCase_rel	Relative frequency of Orpha cases normalized to 100,000 inpatient cases
P6	Tracer Case rel. Frequency	tracerCase_rel	Relative frequency of tracer cases normalized to 100,000 inpatient cases
P7	Missing Mandatory Data Items	im_misg	Number of mandatory data items that are missing
P8	Missing Mandatory Data Values	vm_misg	Number of mandatory data values that are missing
P9	Outliers	vo	Number of outlier data values (vo)
P10	Missing Orphacodes	oc_misg	Number of tracer diagnosis codes where no Orphacode is present
P11	Implausible Links	link_ip	Number of ICD-10-GM Orphacode combinations not found in Alpha-ID-SE
P12	Duplicated RD Cases	rdCase_dup	Number of cases with identical case ID, patient ID, ICD-10-GM code, and Orphacode (if available)
P13	Ambiguous RD Cases	rdCase_amb	Number of RD cases diagnosed using an ambiguous ICD-10-GM/Orphacode link or ICD-10-GM tracer diagnosis

The comparison of two datasets stemming from the same data source PAS allows for additional DQ assessments, considering one dataset as a reference. EXP-DS serves as a reference for this comparative analysis because it is directly exported from the PAS and has not undergone the data transformation and standardization processes evident in FHIR-DS, as illustrated in Fig. 1. We assume that automated pipelines do not add data so that mainly information can be lost at different levels. However, we would like to mention that manual data curation in claims data creation, as shown in Fig. 1, may actually insert and modify data, violating this assumption. Therefore, the following approach should be taken with care.

We define the comparative DQ parameter (Pi_lost) as the difference between the parameter (Pi_EXP) obtained from the direct data export and here defined as reference, and the parameter (Pi_FHIR) obtained from the FAIRified dataset. For example, the parameter P1_lost = P1_EXP - P1_FHIR describes the difference between the number of RD cases found in EXP-DS and FHIR-DS. Due to some parameters referring to missing values, and some to present values, we define Pi_lost as:

$$Pi\_lost=\left\{\begin{aligned}&Pi\_FHIR - Pi\_EXP,\,for\,i=7,8,9 \\&Pi\_EXP - Pi\_FHIR,\,for\,i \in [1,13]\,else\\\end{aligned}\right\}$$

We would further like to note that a Pi_lost > 0 does not always indicate a DQ issue. For example, a reduction of duplicates (P12_lost) or outliers (P9_lost) due to data curation within the FAIRification process might be intentional.

The assessment of DQ was implemented using the self-developed open-source DQ library written in R (dqLib). This R package enables traceable and explainable assessments of clinical DQ, and is published on Zenodo and GitHub [32]. The library provides a set of methods for calculating required DQ metrics and generating specific reports on detected DQ issues [32]. The actual DQ assessment software CordDqChecker provides a FHIR interface and is available as source code or as a Docker container [27]. In this study, we added multi-CPU parallelization which was implemented as a configurable option (see section “Performance analysis’’). The resulting DQ reports encompass two Excel spreadsheets for each dataset. The first spreadsheet – called “DQ Metrics Report’’ – reports the calculated DQ indicators and parameters, while the second sheet – called “DQ Violations Report’’ – lists the detected DQ issues. The generated reports were independently verified by domain experts. If no contradiction can be found the reports are considered as correct. For further reading on the DQ assessment methods and tools, please refer to [22, 23]. Effective DQ assessment should support enhancing both the overall DQ and employed FAIRification processes. If these criteria are met, the developed DQ assessments can be considered effective. Therefore, the ETL processes were adjusted based on DQ findings, and the respective DQ assessments were repeated.

The secondary use of RD data raises specific legal and ethical concerns that surround the code for General Data Protection Regulation (GDPR) and the risk of patient re-identification from the data. Hence, to protect RD data from re-identification, specific details on the detected DQ issues cannot be disclosed. As a solution, we use a technique called k-anonymity principle, which ensures that each patient is indistinguishable from at least k−1 other patients in the dataset. This allows us to present illustrative examples in the “Results” section without revealing any sensitive information about individual patients.

### Performance analysis

We conducted a performance analysis to evaluate the efficiency of the developed software. In this context, we performed DQ assessments using two computers equipped with Intel Xeon Gold 5220s multi-core processors and 16 GB RAM, one computer for each dataset. The assessments were executed using R version 4.2.2.

The FHIR server was operated on a server with an Intel CPU consisting of 48 cores at 2.7 GHz clock speed and 512 GB RAM. We measured the runtime of the DQ assessment on the FHIR-DS with and without parallelization enabled.

### FAIR software assessment

The GitHub repository provides synthetic datasets, a Docker image, and specific instructions [27]. Additionally, we compared the current implementation of the DQ assessment software with the recent recommendations given specifically for FAIR research software, called FAIR4SR principles [33]. The FAIR4RS principles concretize the original FAIR principles for research software. As they are not as well known as the FAIR principles, the principles are provided in the Supplementary File.

## Results

### DQ assessment

Table 4 shows the assessment results of the first six DQ parameters. Notably, from the 1424 RD cases selected by the CORD-MI Tracers, only about half of them (729) were identified through an Orphacode, while only about 11% (165) could be identified by the Alpha-ID-SE Tracers.

**Table 4:** RD-specific DQ parameters evaluated before and after the implementation of FAIRification processes

No.	DQ-parameter	Datasets	
		EXP-DS	FHIR-DS
P1	RD Cases	783	163
P2	Orpha Cases	729	0
P3	Tracer Cases	165	163
P4	RD Case rel. Frequency *	1125	234
P5	Orpha Case rel. Frequency *	1047	0
P6	Tracer Case rel. Frequency *	237	234

* Frequency normalized to 100.000

**Fig. 3:**
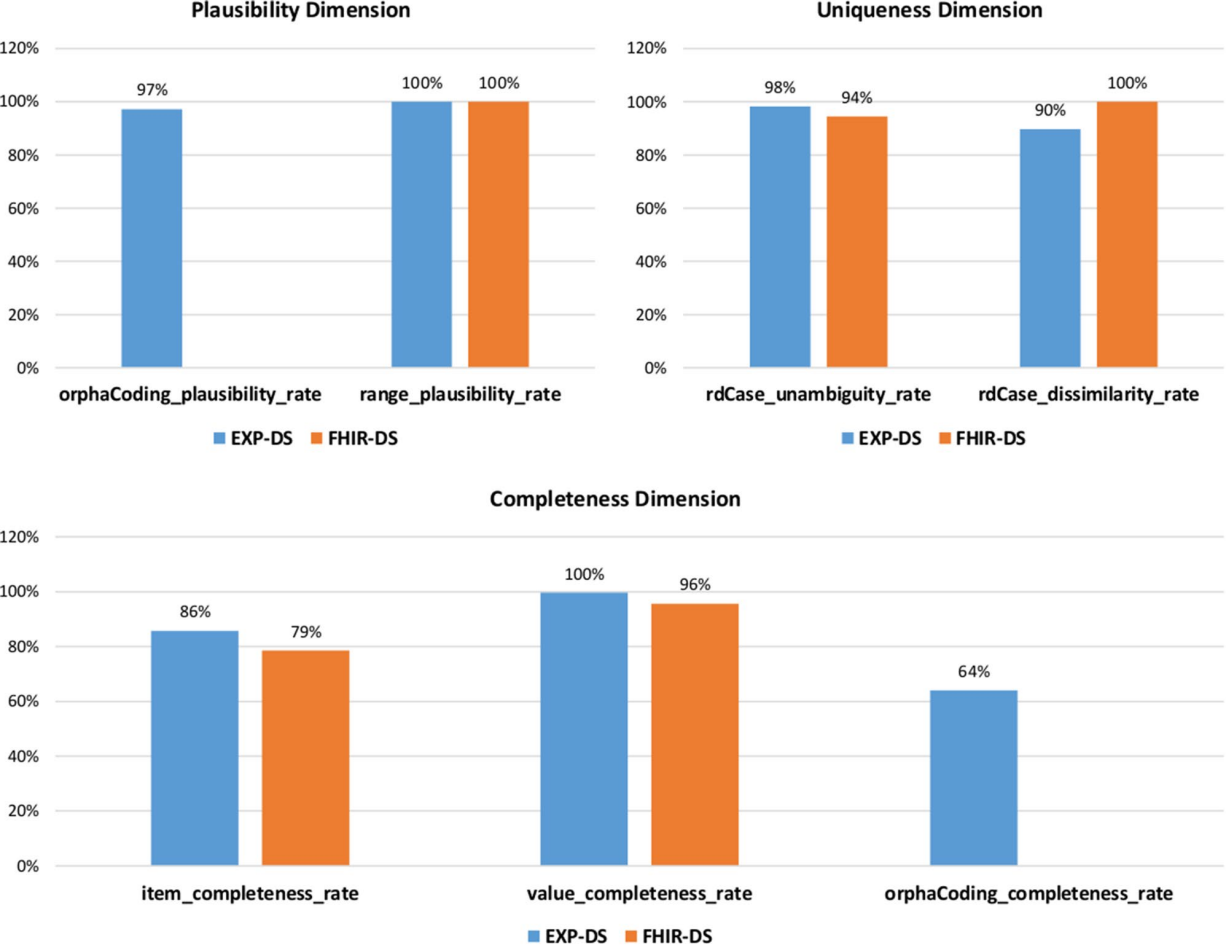
Local DQ assessments using the SAP and FHIR data recorded in 2021. It should be noted that the completeness of data values reached its maximal level after resolving the FHIR mapping issues

Figure 3 shows the resulting DQ indicators for both datasets. Differences between the DQ results from the two different sources can be observed in all DQ dimensions. Only the Range Plausibility Rate (dqi_pl_rpr) scored 100% for both datasets. Another optimal score is found for the Value Completeness Rate in EXP-DS. As no Orphacodes are present, the Orphacode-related DQ indicators show 0% for FHIR-DS. The Item and Value Completeness Rates and the RD Case Unambiguity Rate are lower in the FHIR-DS. The only indicator that is higher in the FHIR-DS is the RD Case Dissimilarity Rate.

**Fig. 4:**
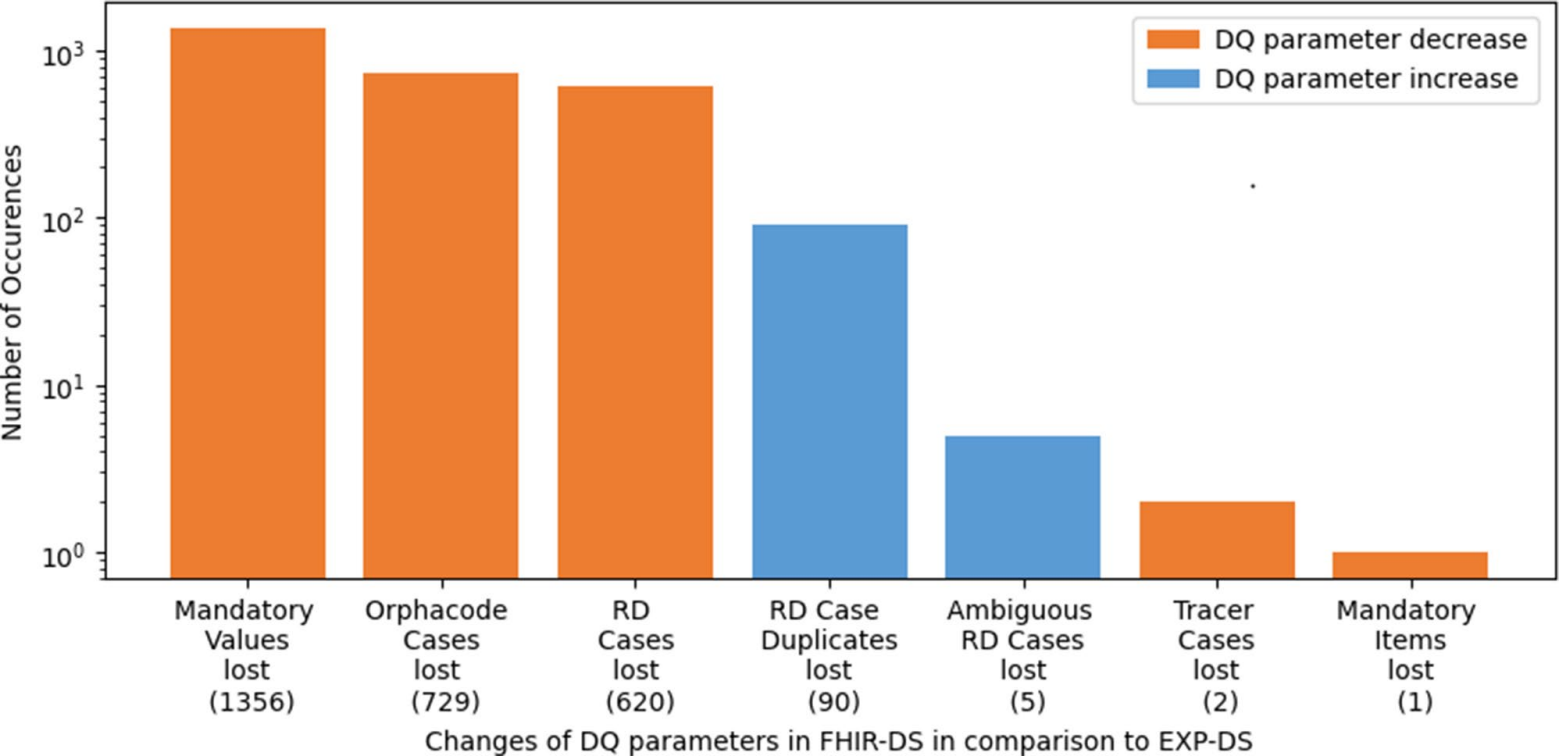
Effect of FAIRification processes on the quality of RD data: A comparative analysis of DQ parameters in two datasets (EXP-DS and FHIR-DS) extracted from the same data source but processed differently

The DQ analysis of the EXP-DS revealed 192 issues that comprised missing data items (2) , missing Orphacodes (65), ambiguous RD cases (14), duplicated RD cases (90), and implausible RD diagnoses (21) . Furthermore, more DQ issues were identified in FHIR-DS, as shown in Fig. 4. This figure illustrates Pi_lost on a logarithmic scale, which visualizes the differences in the number of occurrences for both datasets. It can also be observed that the majority of DQ issues within FHIR-DS belong to the completeness dimension, since more missing values (1356) and items (1) and fewer RD cases (620) were found here. Summing up to 1977 completeness issues were introduced by FAIRification processes. Additionally, 9 ambiguous cases and 2 missing items were detected, resulting in a total of 1988 distinct DQ issues within FHIR-DS. However, we resolved all missing value issues, which represented 68% of all DQ issues.

To illustrate the practical relevance of identified DQ issues without compromising data privacy, we provide anonymized examples of DQ issues for each quality dimension with a frequency that surpasses five. Regarding plausibility, around 29% of detected implausible links were related to RD cases diagnosed using the ICD-10-GM code “G71.2”. According to Alpha-ID-SE terminology, the code “G71.2” encompasses more than 20 potential Orphacodes that represent distinct RDs, but this code is not linked to the Orphacodes found in these implausible pairs. The evaluation of data uniqueness revealed multiple ambiguous RD cases documented with the ICD-10-GM code “F84.2’’, which represents two distinct RDs, namely Rett syndrome (Orpha:778) and Atypical Rett syndrome (Orpha:3095). Concerning data completeness, 26 missing Orphacodes were identified in EXP-DS for RDs documented with the ICD-10-GM codes “M34.1” and “D86.1”. Furthermore, the FAIRification processes introduced new completeness issues within the FHIR-DS, such as missing data values. The “Discussion’’ section provides further details on the underlying causes and factors contributing to the identified DQ issues.

### Performance analysis

The runtime of the DQ assessment on the CSV file of EXP-DS took about 19 s.

The processing of the DQ assessment on FHIR-DS – which included communication with the FHIR server – took 12.52 min without parallelization. The version with enabled parallelization required about 7 s to complete the DQ assessment.

### FAIRification of research software

For a description of the individual principles, please refer to the Supplementary File and the original publication [33]. Both the developed software and the underlying library were published as open source on the online version control system GitHub. As a shared code repository, they have already been implemented in accordance with five FAIR principles: F1 (unique identifier), F1.2 (versioning), A1 (accessible), A1.1 (free and open protocol), and R1.2 (provenance). Both repositories have persistent digital object identifiers (DOIs), as specified in F1, and support contributor authentication when committing code changes, as required by A1.2 (authentication procedure). F1.1 (modularity) is fulfilled by the separate repositories for the generic library (dqLib) and the current assessment software (CordDqChecker). F2, F3, and F4 are satisfied by providing metadata in a standardized format that facilitates the citation of the software used in a searchable form. Specifically, dqLib is archived on Zenodo, which provides rich metadata. Additionally, the code repository provides separate files that contain metadata to describe the assessment software CordDqChecker and its dependencies, including a Docker image and configuration file. I.1 (community standard data handling) is addressed by CSV import, the FHIR interface, and an XLS-based output. I1.2 (references to other objects) is addressed by references to the used FHIR profiles and reference lists. R1 (software attributes) is satisfied because detailed information about the author and development history is included in the GitHub repositories for both the library and the DQ assessment tool. R1.1. (license) is also fulfilled for both software tools. As other libraries are included with the standard method R provides, R2 (qualified references to other software) is also implemented. Finally, R3 (domain-relevant community standards) is fulfilled, as R is a very common open-source programming language in biomedical statistics. As a result, 16 of the 17 principles for FAIR research software were fulfilled.

## Discussion

### DQ issues found

Most DQ issues detected in this study were related to the completeness dimension. Our assessments revealed a total of 2044 completeness issues across both datasets. These DQ issues included missing data values (1356), missing RD cases (620), and missing data items (3) in FHIR-DS, as well as missing Orphacodes (65) in EXP-DS. The majority of the reported completeness issues were missing mandatory data values, partly attributable to a non-conformant mapping with the MII-CDS standard. For instance, mapping the primary and secondary diagnoses to the FHIR specification of the MII-CDS led to new issues related to data completeness, which were subsequently resolved. As mentioned previously, the detected missing RD cases (see Fig. 4) were attributable to Ophacodes not being part of the § 21 dataset in 2021 and therefore not available in FHIR-DS, even though Orphacodes were documented in the HIS. Hence, Orphacoding completeness and plausibility could not be assessed for the FHIR-DS. Moreover, the MII-CDS did not define Orphacode as a mandatory data item. Therefore, the assessment of item completeness (see Fig. 4) did not consider the lack of Orphacodes as a DQ issue. Two missing data items were linked to sensitive information related to the patient’s address, which was not maintained in either dataset due to privacy regulations. These privacy regulations are especially strict for RDs as the re-identification risk is high. It should be noted that both data items have been set as optional in a subsequent version of the MII-CDS. The third missing data item resulted from the incorrect application of FHIR guidelines for mapping the MII-CDS.

Overall, the findings show the effects of the implemented FAIRification processes on DQ using a comparative analysis of DQ issues identified in EXP-DS and FHIR-DS. Nearly all (99%) of the identified DQ issues (1988) in FHIR-DS were attributed to challenges in the FAIRification processes. Notably, completeness was the primary DQ dimension affected in this study, with completeness issues largely stemming from the transformation to claims data based on § 21, data privacy issues, and issues related to MII CDS conformity and interpretation. However, most of these completeness issues were subsequently resolved by refining the MII-CDS conformity.

Our study indicates that DQ is influenced, among other factors, by decisions made during software development, e.g., how to implement a common data schema like the MII-CDS and the specific features of the data source, similar to issues reported by Spengler [14]. While Spengler et al. [14] only analyzed the quality of hospital claims data during ETL processes, our study evaluated the quality of two RD datasets, both originally derived from the same PAS, but involving different transformation processes, selection criteria, and output formats. Our findings indicate that data transformation and adherence to standards, such as the MII-CDS, can substantially affect DQ. Therefore, evaluating DQ especially in the context of data standardization efforts across many locations, as was done in the MII, is essential to maintain high-quality EHR data, particularly in the context of RDs where low data availability is common.

Identifying and understanding the factors that influence DQ are vital for mitigating DQ issues in clinical documentation and are especially valuable when it comes to alignment of Orphacoding practice with the requirements by law, defined in the Alpha-ID-SE. Our analysis revealed that the key factors contributing to DQ issues within the data capturing processes for RDs were challenges in the semantic annotation of related diagnoses, along with the lack of an efficient software solution aligned with the Alpha-ID-SE terminology to notify users of potential documentation errors. As shown above, cases with ambiguous diagnoses are common in RD documentation. This may be explained by the nature of RDs where diagnostic data are often ambiguous [23]. Such documentation issues even make the majority of RDs invisible in HISs, as reported by Aymé et al. [17]. Ambiguity issues in diagnostic data are particularly critical because they impede a comprehensive understanding of related clinical data and may affect the outcomes of clinical studies. Therefore, unambiguous RD coding is vital for enhancing the quality and visibility of RD cases in HISs. In contrast to our initial analysis [22], this study offers a deeper understanding of the factors influencing DQ and the related issues. We also employed newer data and expanded our methods according to the FAIR principles.

Although the FAIRification of RD data presents challenges as previously reported, we are confident that the benefits of achieving FAIR data justify the added complexity and efforts required. As described in the introduction, due to these efforts the secondary use of clinical routine data including RDs is now available for researchers worldwide through the FDPG.

### DQ metrics

The employed indicators generally yielded considerable results of over 60% on the EXP-DS across all dimensions of DQ, in a field where acquiring high-quality data is often challenging [34]. All completeness indicators performed better on EXP-DS than on FHIR-DS for the reasons described above (see Fig. 3). The initial DQ analysis led to an improvement in the conformity of the FHIR-DS with the FHIR standard specification of MII-CDS, showing the direct benefits of DQ assessment, especially in the context of standardization efforts as realized in the MII. This indicates that using DQ indicators motivates action [35] and drives DQ improvement processes.

Compared with our previous study with data from 2020, Orphacoding completeness has shown better results on the EXP-DS from 2021 employed in this study [22]. Both RD Case and Orpha Case frequencies (see Table 4) have doubled in 2021 when compared to the former results. This increased level of Orphacoding completeness may explain the rise (around 57%) in the visibility of RD cases compared to our initial results. Consequently, our findings provide evidence supporting the hypotheses of Rath et al. [36] that the use of Orphacodes is expected to increase the visibility of RDs in the HIS. Furthermore, the normalized DQ parameters used in this study can also be useful prospectively for comparing RD visibility across multiple hospitals.

In contrast to completeness indicators, our study has shown an improved dissimilarity indicator (dqi_un_cdr) in FHIR-DS. The second uniqueness indicator Unambiguity Rate of RD Cases (dqi_un_cur) achieved strong results (> 94%) in both datasets, which was expected due to the selection of an RD study population according to RD-specific ICD-10-GM codes. Our methodology successfully computed the Unambiguity Rate using available ICD-10-GM codes, even though the FHIR-DS did not contain Orphacodes. This demonstrates that our methodology is adaptable to the absence or presence of Orphacodes as an approximation of the number of RDs can still be given using ICD-10-GM codes alone. We would like to note that the Unambiguity Rate of RD Cases only considers cases that are unambiguously RDs – the used ICD-10-GM code may subsume more than one RD, but no common diseases. Our assessments showed that this indicator was 4% lower in the FHIR-DS due to the lack of Orphacodes. Although the analyzed cases were selected using diagnoses defined by clinical experts, namely the CORD-MI Tracers, this finding shows the limitation of the used ICD-10-GM codes and the necessity of unambiguous semantic annotations to enable an accurate interpretation of these RD cases. In line with Denton et al. [34], our findings affirm the importance of standardized semantic annotation for ensuring high-quality RD data and supporting clinical research on RDs.

Noteworthy is the plausibility dimension, with rates exceeding 97%. A total of 21 DQ issues were detected related to the RD diagnoses. Special characters contributed to 38% of the identified plausibility issues. It is important to consider the design aspects of the information systems used to avoid such plausibility issues [37].

### Methods

The developed DQ assessment provides useful metrics and tools for establishing an interactive and iterative DQ improvement process. Our findings have shown the efficiency and effectiveness of our methods in identifying DQ issues like cases with ambiguous or implausible diagnoses. The generated DQ reports were validated independently by domain experts from the University Hospital Tübingen and enabled us to understand the causes of detected DQ issues and enhance the quality of RD data. Following recent studies on research infrastructure in the medical and biological sciences [38–40], our technical solution also adheres to the FAIR guiding principles. As a result, we shared the developed tools, metadata, and synthetic data with the scientific community to enhance the quality of RD data and support clinical research. This approach contributes to the sustainability and reusability of DQ assessments. Furthermore, our DQ concepts are aligned with the harmonized DQ terminology proposed by Kahn et al. [20], which was implemented in various research studies [14, 41]. On the other hand, our study emphasizes the necessity of considering specific requirements for DQ assessments, as indicated by Tute et al. [42]. This approach, however, does not support FHIR and lacks DQ indicators for evaluating the quality of RD data. Additionally, this approach also lacks coverage of important aspects of DQ such as semantic uniqueness and syntactic uniqueness, which are essential for enabling the secondary use of RD data as shown above. Our methodology offers a key advantage over previous approaches [19–21, 41–43] by considering domain-specific requirements alongside parallel computing and FAIR principles.

Overall, performance analysis and optimization techniques showed that the parallelization of FHIR requests can speed up the DQ analysis by two orders of magnitude. Regarding generalizability, the methodology proposed in this study can be used to assess the quality of RD data across multiple hospitals, as shown in the final report of CORD-MI [44]. Notably, generic indicators, such as the Range Plausibility Rate and Value Completeness Rate, can also be reused for different use cases and datasets that follow the MII-CDS specifications. Moreover, the proposed methods can be generalized to common diseases as well. The first results from leveraging these methods in cardiovascular diseases are available on GitHub [45].

### Limitations

Our study comes with certain limitations. The identification of all RD in claims data is a challenging task, even if Orphacoding according to Alpha-ID-SE is mandatory in Germany since 2023.

In the investigated dataset using the ICD-10 code included in claims data, this task can only be done exemplary with RD-specific ICD codes and thus a somewhat arbitrary subset of RDs. Alpha-ID-SE 2022 was used as a reference for assessing the quality of diagnostic data on RDs, but this semantic resource did not cover all Orphacodes provided by the Orphanet nomenclature. Nevertheless, the version 2022 of Alpha-ID-SE used for this analysis includes the most relevant RDs and is updated annually with new RD diagnoses to achieve full coverage of the disorders listed in Orphanet. Our DQ assessments used a tracer diagnoses list created by automatic analysis of the Alpha-ID-SE, which depends on consistent Orphacode annotation for all relevant diagnostic terms within the Alpha-ID-SE. However, consistent annotation of all diagnostic terms related to RDs is challenging, and consequently, the 2022 version of Alpha-ID-SE remains incomplete. In effect, only about 10% of the cases included based on the CORD-MI Tracers were completely annotated in Alpha-ID-SE and therefore identified as Tracer cases, although this standard encompasses 303 Alpha-ID-SE Tracers, while only 143 CORD-MI Tracers are defined. It should be noted that the absence of an Orphacode for a diagnostic term within the Alpha-ID-SE does not currently allow for concluding that this term refers to a common disease. Nevertheless, completeness of Orphacoding can be assessed with certainty and in accordance with regulatory requirements for all Alpha-ID-SE Tracers. In this context, the unevaluated remaining cases may be further assessed by Alpha-ID-SE experts, who can determine whether they represent common diseases or RDs that should be incorporated into future revisions of Alpha-ID-SE. This reflects an ongoing process of refining and expanding the Alpha-ID-SE over time.

Furthermore, it is important to note that the report on DQ issues contains sensitive information related to patients and medical diagnoses, which cannot be included in this paper due to data privacy regulations. An exemplary report is however presented in a previous publication [23].

Another limitation is that we did not have direct access to PAS data, so source validation was not feasible. Additionally, manual data curation steps are a black box and not all DQ issues can be attributed to a certain cause.

## Conclusion and outlook

Our analysis shows the importance of evaluating the impact of data selection, transformation, and standardization in FAIRification processes on DQ, as these processes can introduce major DQ issues. These measures are necessary for maintaining high-quality FAIR data. In line with previous studies [1, 46–48], we have shown that EHR-based research data may suffer from various types of DQ issues, as reported above. Notably, completeness indicators have proven essential for evaluating the quality of RD data, as most identified DQ issues are related to the completeness dimension. Moreover, the findings indicate that the CORD-MI Tracers were unable to fully address the ambiguity issues detected within the study data. Such issues raise concerns about the quality of RD data, even when the study population is selected based on ICD-10 diagnoses defined by clinical experts.

Different DQ issues can occur depending on the features of the data source and the data transformation and standardization processes involved. These findings demonstrate that our methodology is adaptable to different scenarios and can be applied in a wide range of contexts. Specifically, our study shows that the absence of Orphacoding does not preclude the analysis of DQ for RD research. Therefore, data sources that do not contain Orphacodes may yield DQ sufficient for RD research in some instances – our tools allow such an assessment for specific studies taking into consideration the available data and research question.

Our assessments have substantially supported the understanding and improvement of the described FAIRification processes and overall DQ. We have found that DQ issues such as ambiguity and plausibility issues are particularly complex as they may arise in several steps within the data ingestion workflow. It should be noted that these issues can typically make the reuse of affected data for clinical research difficult or even impossible. The insights acquired from this work may also be useful for similar studies. Throughout this study, we have shown the usefulness and efficiency of developed DQ assessments. The employed harmonized DQ concepts and normalized indicators allow for the comparison of DQ results across multiple studies or hospitals. Therefore, we recommend conducting cross-institutional DQ assessments on RD data. For instance, we have been able to successfully roll out our DQ assessments across multiple hospitals within the CORD-MI research network [44]. Furthermore, we intend to expand our implementation to offer more advanced visualization and anonymization methods as well as to cover additional FHIR profiles of the MII.

As the CORD-MI consortium is embedded in the European networks on RDs, such as the European Reference Networks and the European Rare Diseases Research Alliance [49, 50], the translation of the methods to broader use is envisioned as a next step. The developed methods support in particular current initiatives to use EHR as a data source for RD registries and studies. According to previous studies on RDs [34, 51], AI algorithms have the potential to support RD drug development. However, the effectiveness of these algorithms depends on the quality of the data used. Ambiguous or implausible EHR data may hinder the application of such tools in the field of RDs, where low data availability is common. Consistent with Denton et al. [34], we emphasize the necessity of high-quality RD data and underline the importance of assurance measures to enable the effective application of AI algorithms in the field of RDs. Our methodology can help ensure that EHR data is of high quality and thereby support the application of AI algorithms in clinical research on RDs.

## Supplementary Information

Below is the link to the electronic supplementary material.

Supplementary File 1

## Data Availability

The datasets analyzed during the current study are not publicly available due to patient data privacy, but exemplary datasets in CSV and FHIR formats are provided on GitHub at https://github.com/KaisTahar/cordDqChecker/tree/bmc_dqTools. The original datasets are archived according to Good Scientific Practice. All tools and detailed instructions used in this study are also openly accessible in the same GitHub repository.
