# Supplementary material for: Sharing EHR data of patients with rare diseases for research - The role of quality assessments in a national federated research data infrastructure
Source: BMC Med Inform Decis Mak. 2026 Mar 2;26:60. doi: 10.1186/s12911-025-03153-x (PMC12955070; doi:10.1186/s12911-025-03153-x)
Supplement: Supplementary file 1 [file 12911_2025_3153_MOESM1_ESM.docx]

**FAIR Principles for Research Software**

**F: Software, and its associated metadata, is easy for both humans and machines to find.**

F1. Software is assigned a globally unique and persistent identifier.

F1.1. Components of the software representing levels of granularity are assigned distinct identifiers.

F1.2. Different versions of the software are assigned distinct identifiers.

F2. Software is described with rich metadata.

F3. Metadata clearly and explicitly include the identifier of the software they describe.

F4. Metadata are FAIR, searchable and indexable.

**A: Software, and its metadata, is retrievable via standardised protocols.**

A1. Software is retrievable by its identifier using a standardised communications protocol.

A1.1. The protocol is open, free, and universally implementable.

A1.2. The protocol allows for an authentication and authorization procedure, where necessary.

A2. Metadata are accessible, even when the software is no longer available.

**I: Software interoperates with other software by exchanging data and/or metadata, and/or through interaction via application programming interfaces (APIs), described through standards.**

I1. Software reads, writes and exchanges data in a way that meets domain-relevant community standards.

I2. Software includes qualified references to other objects.

**R: Software is both usable (can be executed) and reusable (can be understood, modified, built upon, or incorporated into other software).**

R1. Software is described with a plurality of accurate and relevant attributes.

R1.1. Software is given a clear and accessible license.

R1.2. Software is associated with detailed provenance.

R2. Software includes qualified references to other software.

R3. Software meets domain-relevant community standards.
